# Internet of Unmanned Aerial Vehicles—A Multilayer Low-Altitude Airspace Model for Distributed UAV Traffic Management

**DOI:** 10.3390/s19214779

**Published:** 2019-11-03

**Authors:** Nader Samir Labib, Grégoire Danoy, Jedrzej Musial, Matthias R. Brust, Pascal Bouvry

**Affiliations:** 1 SnT—University of Luxembourg, L-4364 Esch–Sur–Alzette, Luxembourg; matthias.brust@uni.lu (M.R.B.); pascal.bouvry@uni.lu (P.B.); 2 FSTC—CSC ILIAS, University of Luxembourg, L-4364 Esch–Sur–Alzette, Luxembourg; 3 Institute of Computing Science, Poznań University of Technology, 60–965 Poznań, Poland; jedrzej.musial@cs.put.poznan.pl

**Keywords:** Internet of Things, traffic management, autonomous unmanned aerial vehicles, low-altitude airspace, multilayer networks

## Abstract

The rapid adoption of Internet of Things (IoT) has encouraged the integration of new connected devices such as Unmanned Aerial Vehicles (UAVs) to the ubiquitous network. UAVs promise a pragmatic solution to the limitations of existing terrestrial IoT infrastructure as well as bring new means of delivering IoT services through a wide range of applications. Owning to their potential, UAVs are expected to soon dominate the low-altitude airspace over populated cities. This introduces new research challenges such as the safe management of UAVs operation under high traffic demands. This paper proposes a novel way of structuring the uncontrolled, low-altitude airspace, with the aim of addressing the complex problem of UAV traffic management at an abstract level. The work, hence, introduces a model of the airspace as a weighted multilayer network of nodes and airways and presents a set of experimental simulation results using three UAV traffic management heuristics.

## 1. Introduction

Characterised as a mega-network of heterogeneous technologies and standards, Internet of Things (IoT), broadly refers to a network of uniquely addressable, interconnected objects, built on standard communication protocols whose point of convergence is the internet [1]. The fundamental idea behind IoT lies in connecting, both, virtual and physical everyday objects, thus enabling a wide array of services that were otherwise deemed unfeasible to be realised.

**Figure 1:**
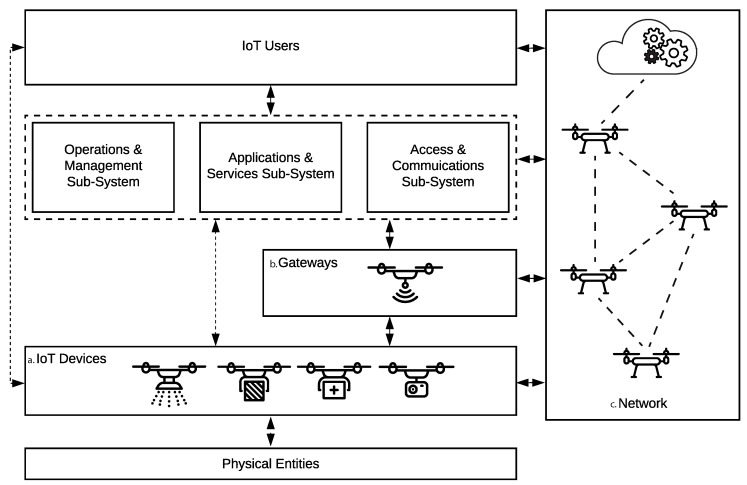
Role of unmanned aerial vehicle (UAV) within Internet of Things (IoT) as: (**a**) smart terminal devices that interact with the physical world; (**b**) aerial base stations and gateways; (**c**) communication network connected to IoT cloud.

The widespread of IoT has, therefore, motivated the advancement in information communication technologies, catalysing the development and integration of new devices to this ubiquitous network. One promising category of devices that recently found its way into IoT, is Unmanned Aerial Vehicles (UAVs). The miniaturisation of lightweight, low energy consumption, wireless sensors has led to the rapid advancement in UAV technologies. In turn, promising new means of efficiently collecting and transmitting data as smart terminal devices capable of interacting with the physical world for a magnitude of IoT applications. Some predominant examples include smart agriculture [2,3], smart logistics [4], smart healthcare [5] and monitoring, surveillance and disaster management [6,7,8,9]. Nevertheless, UAVs promise a pragmatic solution to the limitations of existing terrestrial IoT infrastructure that, in some cases, would not be economically feasible nor sufficient to guarantee communication coverage with an acceptable level of quality [10]. Hence, making aerial technologies, such as UAVs a promising solution where they can contribute to overcoming such limitations by offering wider coverage, better availability and higher resilience when equipped with the appropriate sensory payload [11,12]. Figure 1, based on the International Organisation for Standardisation (ISO) and the International Electrotechnical Commission’s (IEC) IoT reference architecture published in ISO/IEC 30141 [13], illustrates some examples of potential roles for UAVs within IoT.

**Figure 2:**
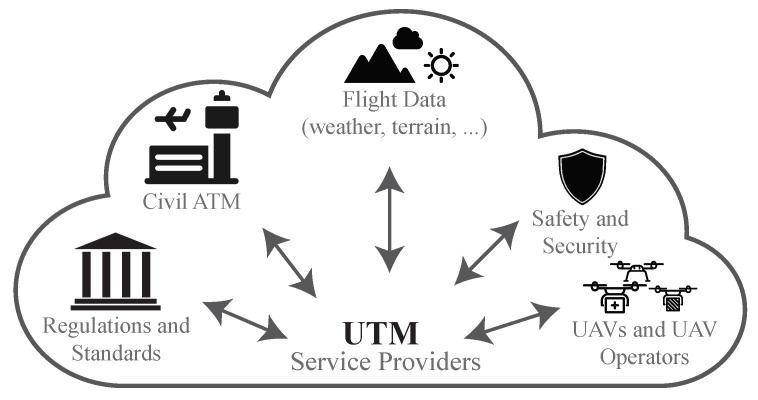
UAV traffic management (UTM) facilitating communication between main stakeholders [22].

As research and industry continue to find more uses for UAVs [14], it is reasonable to envision a near future where heterogeneous swarms of UAVs would dominate the low-altitude airspace, operating beyond direct line of sight, hence, making their safe operation and management a critical research challenge. One viable solution is a dedicated UAV Traffic Management (UTM) system [15], an infrastructure building upon IoT concepts, such as its layered design, to complement conventional Air Traffic Management (ATM) by facilitating data exchange between UAVs as well as different stakeholders, as illustrated in Figure 2. However, in comparison to Air Traffic Management (ATM), UAV Traffic Management (UTM) introduces new research challenges with UAVs’ higher degree of mobility and energy limitations. Recently becoming an important research topic, with the expected large airspace traffic demand, dynamic geo-fencing and intrusion detection requirements, the currently under-development constructs, like NASA UTM [16] and EU U-Space [17] to name a few, will eventually face limitations in scalability and resilience due to their ATM–comparable centralised architecture. On the contrary, a distributed UTM relying on local decisions and ad-hoc UAV-to-UAV and UAV-to-infrastructure communications would reduce latency by allowing UAVs to exchange the more frequent awareness messages directly as explained in Reference [15], utilising paradigms such as multi-access edge computing [18]. Hence, achieving scalability and better resilience [19].

A mandatory first step towards a distributed UTM is to design and model the low-altitude airspace as its structure will have a significant role in air traffic management. This is emphasised in Referebces [20,21] where authors investigate the impact of the airspace structure and capacity for traffic densities.

This work builds on the foundations laid in our previous work [22] where we addressed the aforementioned, complex problem of UAV traffic management at an abstract level by proposing a structure for the uncontrolled low-altitude airspace; presented as a weighted multilayer network of nodes and airways. The main contribution of this paper, therefore is extending [22] by:

- emphasising on the role of UAVs within IoT;
- providing a broader state of the art analysis;
- extending the UTM model description with UAV communication;
- considering more realistic experimental scenarios.

The remainder of this work is structured as follows. Section 2 discusses the state of the art on multilayer networks, UAV traffic management and distributed path planning. Section 3 describes the proposed multilayer low-altitude airspace model followed by a corresponding optimisation problem and resolution approaches in Section 4. Section 5 explains our experimental setup and results analysis. Finally, Section 6 concludes the work and presents future research directions.

## 2. State of the Art

This section first presents the related work in multilayer networks as the groundwork on which our model is based, followed by a discussion on recent UTM developments and autonomous robot path planning. At the end of each subsection we emphasise our contributions to the literature.

### 2.1. Multilayer Networks

One particularly useful way to study complex systems is by analysing the networks that encode the interactions among the system’s elements. However the complexity of some real systems is such that it is not possible to study them as single layer networks. To account for this complexity, a more general framework, known as multilayer networks is considered. Over the recent years, research in physics and computer science developed different notions and models for complex networks referred to as networks of networks [23], multilayer social networks [24] and interconnected networks [25] to name a few. Literature provides many applications for such systems in ecology [26], biology [27], economic applications [28] and game theory [29] but what interests us most are those addressing transportation [30].

Transportation systems are one distinct example of systems where the multilayer formulation arises in a natural way [30] as there can be multiple modes of transport between given locations. This can be represented as a multilayer network where each layer is a representation of one mode of transportation forming an already complex network. Different segments, later referred to as *paths*, can have very different properties such as *allowable velocity*, *energy consumption* or *traffic capacity* and it is thus necessary to distinguish each of them when studying the whole system [30]. A similar multilayer representation is used in aerial transportation systems such as in Reference [31] where the authors built the European air transport multilayer network having each network layer represent an airline. Similarly, References [32,33,34] analyse, respectively, the Greek and Chinese airline transportation networks using the same aforementioned framework.

Our main contribution is expanding a similar methodology to represent the structure of the low-altitude airspace—Class G—as a multilayer network which, to the best of our knowledge, has never been proposed in the air traffic management literature.

### 2.2. UAV Traffic Management

With the evolution of wireless sensor networks and the potential uses of IoT–enabled UAVs as smart terminal devices in a magnitude of applications, it is important to acknowledge and address the legitimate challenges of privacy, airspace management and safety, that will accompany their integration into Class G airspace over cities [14].

In order to address such pressing issues, Standard Development Organisations (SDOs) and other regulatory bodies have recently established working groups to lay down the foundations to accelerate the development of a new dedicated infrastructure for UAV management [35]. Starting with operator control, vehicle identification and control and, finally, traffic flow control, the envisioned infrastructure should allow seamless integration of heterogeneous systems and facilitate the data exchange between various stakeholders. Conflict detection and resolution, localisation & tracking and scheduling are some among many of the key function a UTM must offer.

From vehicle identification and control, conflict detection and resolution, localisation and tracking to scheduling, the envisioned infrastructure should allow seamless integration of heterogeneous systems and facilitate data exchange between various stakeholders. Supported by the foundations being laid down by Standard Development Organisations (SDOs) recently established working groups [35], research institutes and companies have recently proposed multiple UTM projects. Spearheaded by NASA Ames Research Centre in close collaboration with the Federal Aviation Administration (FAA) and over 125 industry partners [16], literature provides some constructs and architectures as part of on-going projects. Some of the most notable government led initiatives include U-Space project by the European Commission lead by the European Aviation Safety Agency (EASA) [17], China’s Civil UAS Operation Management System (UOMS) [36] the Japanese UTM [37] in collaboration with the private sector. Additionally, over the recent few years, multiple platforms were proposed by commercial industry. Deployed in over 9 countries across Europe, Asia and the United States of America, one platform that stands out is AirMap’s UAS Traffic Management [38], building on their widely adopted ATM platform. AirMap facilitates the collaboration between flight operators, industry, governments, 7 Standard Development Organisations (SDOs), 4 regulatory bodies including EASA and FAA. The interested reader can find an exhaustive list of commercial concept architectures and constructs in Reference [39] and a regularly updated map of international UTM implementations and test sites in Reference [40].

While the proposed systems offer a viable solution, such centralised systems will not be able to cope with the highly dynamic nature of the UAV traffic networks, let alone the dynamic geo-fencing, intrusion detection and communication challenges. To this extent, we envision a distributed UTM where UAVs dynamically plan their paths based on local information and decisions while optimising ad-hoc communications. This in turn would allow better scalability and resilience of the system [15,19].

### 2.3. Distributed Path Planning

In all the aforementioned promising applications, in order for UAVs or any other autonomous mobile robots, to perform their tasks, efficient and collision-free path planning becomes a necessity. Path planning for robotic applications is a research topic that has been actively studied over many years. However, literature have mainly focused on 2-dimensional (2D) and 2.5-dimensional (2.5D) methods [41], while approaches for UAVs, underwater vehicles and other highly mobile autonomous robots requiring 3-dimensional (3D) path planning, remain less explored.

Commonly used UAVs can be categorised as non-holonomic mobile robots [42] as the degree of their controllable actuators is less than their degree of freedom in the space which they operate; therefore, path planning optimisation adds further complexity in comparison to holonomic systems. In order to address such challenges, researchers divide path planning into two main subsystems; a global path planning subsystem complemented by a lower level addressing collision avoidance. The latter being out of the scope of this work, the interested reader can find various approaches and algorithms in Reference [43].

With focus on global path planning of UAVs, Yang et al. in Reference [44] provide a thorough survey of successful UAV 3D path planning algorithms found in literature. Additionally, the authors analyse and categorise the algorithms into sampling-based, mathematical model based, node-based and bio-inspired algorithms; out of which, we pay particular attention to the latter two, provided our proposed model of Class G. In addition to the well–known Dijkstra and A* algorithms, Likhachev et al. in Reference [45] propose an anytime heuristic search algorithm that improves on classical A* by ensuring that a robot has at least a sub-optimal path at any given time. The authors then develop further on this and propose [46], a heuristic-based re-planning method (AD*) relying on an anytime dynamic A* algorithm to continuously improve its solution within a predefined time frame as well as allow for re-computation of the path when information is updated. Another approach is the Lazy Theta*, proposed in Reference [47], building on the Theta* algorithm, this search method is not constrained to the topology of first hop neighbours in a multilayer network, in turn offers an improvement to classical A*. While this category of algorithms can find optimal paths through decomposing networks, they are not ideally suited for complex environments; hence, researchers rely on bio-inspired algorithms. From Particle Swarm Optimisation, Genetic Algorithm, Artificial Bee Colony and Bacterial Foraging Optimisation, to list a few, literature provides many bio-inspired optimisation algorithms for 3D path planning [41].

In contrast with the majority of the aforementioned heuristics, our algorithm should not only focus on optimising distance and time but should be able to optimise travel time while taking into consideration energy limitations, inspired by energy-aware routing in wireless sensors networks [48]. Therefore, we rely on our previous work in Reference [49] and on the work presented in Reference [50] on Inverted ACO for vehicle traffic management, to adopt a pheromone guided greedy heuristic in order to evaluate UAV traffic behaviour in the proposed model.

## 3. Multilayer UTM Model

This section encompasses our main contributions. We firstly describe the airspace model including key terminology, followed by a formal description of the network model and an illustrative operational example.

### 3.1. Class G Airspace Multilayer Model

The International Civil Aviation Organisation (ICAO) [51] divides the world’s navigable airspace into seven, three dimensional segments, represented by the first seven letters from the ISO basic Latin alphabet. All segments are controlled and regulated by Air Traffic Controllers (ATCs) except for the lower-most one, known as Class G. The latter ranges from 0 to 700 ft above ground level and remains uncontrolled [52] except in the proximity of published airports.

**Figure 3:**
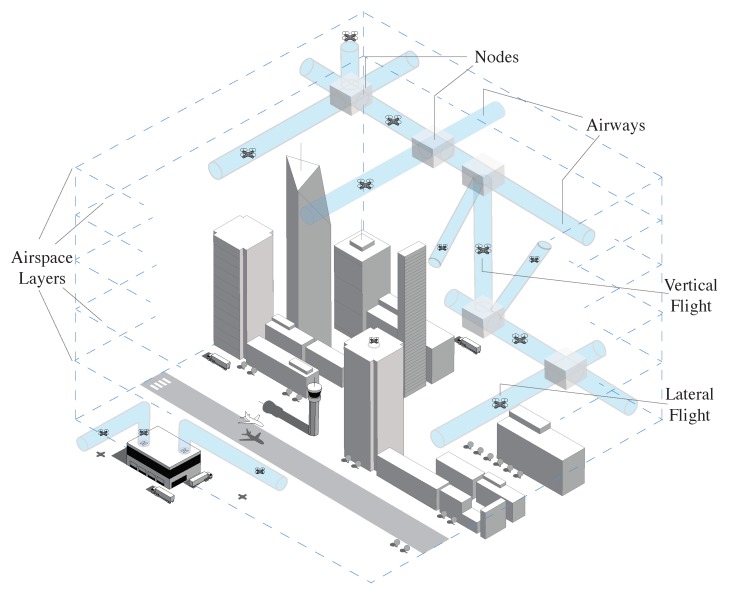
Proposed multilayer UTM model of the Class G airspace [22].

**Figure 4:**
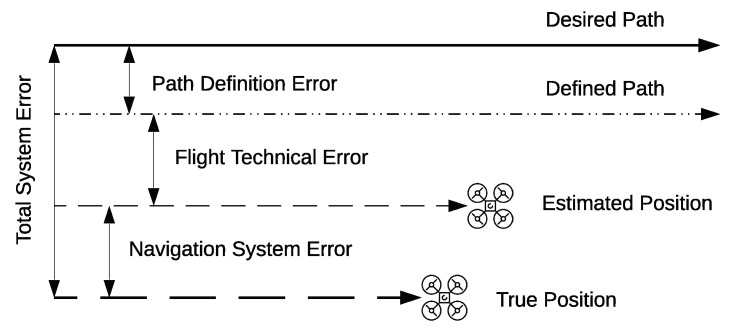
Total System Error of a UAV [22].

In our proposed model, illustrated in Figure 3, we further divide the Class G airspace into horizontal segments, referred to as layers, at different operational altitudes with separation allowing safe UAV flight. This extends a variation of the hemispheric rule [51] to Class G, where the separation between layers is guided by the *Containment Limit (CL)* of the largest UAV allowed in Class G. The CL of an aircraft is explained by ICAO as the volume with a 95% probability the aircraft is within, at any time of its stated position, both horizontally and vertically [53]. This can be derived from the Total System Error (TSE), illustrated in Figure 4. The TSE is the difference between the true position and the position on the desired/assigned flight paths of a UAV. It is the vector sum of the Navigation System Error, the Flight Technical Error and Path Definition error. The TSE defines the accuracy of a navigation system.

**Figure 5:**
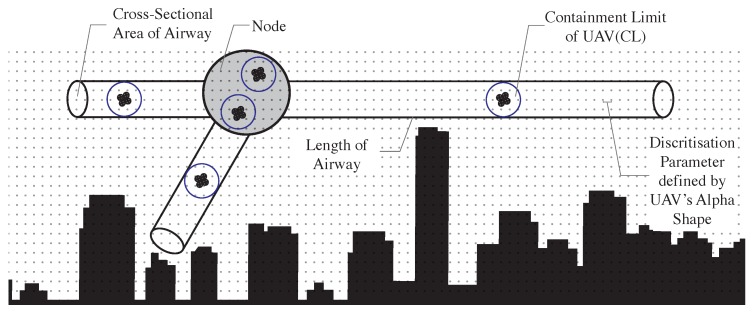
Airways and Nodes in proposed UTM model [22].

Following the approach explained in Reference [54], a city’s elevation map can be discretised using a topological analysis into a data-set of static-obstacle-free points within the different layers. This is key for structured airspace design and path planning. The level of detail of the discretised airspace is defined by the volume representing the UAV (alpha shape) as explained in Reference [55]. The resulting volume of obstacle-free space is referred to as the *airspace* which is the shared resource that is utilised by the UAVs. The latter comprises of *Airways* and *Nodes*, airways being corridors connecting nodes within a layer (horizontal) or between layers (vertical or diagonal). Airways allow UAVs to fly without direct communication with the UTM, guided only by the rules of the airway (velocity limits, flight headings and maximum traffic capacity) and information exchanged between UAVs through ad-hoc communication. Airways’ cross-sectional size is defined by the UAVs’ CL, while their lengths is defined by the segment’s static-obstacle-free space as well as airway-intersections, referred to as nodes (cf. Figure 5).

Within nodes, UAVs are allowed to change their *Flight Mode*. In our model, three main flight modes are considered, *lateral flight*, *vertical flight* and *hovering* for multirotor UAVs. We finally define a *Path* as a complete route from origin to destination, through different nodes and airways. Additionally, in our model, the different airspace layers allow different velocity ranges, that increase with altitude. This is supported by the argument that higher altitudes contain less static obstacles [54] and hence, longer airways are possible. UAVs rely on ad-hoc communication to exchange dynamic traffic information such as their flight velocities and airway traffic density. This in turn reduces latency and allows UAVs to make local routing decisions through the airspace eliminating the need for continuous direct communication with a centralised UTM [15].

### 3.2. Multilayer Network Model

We propose to model of the Class G airspace as a multi-weighted multilayer network, $M_{ClassG}$, where:

$$M_{ClassG}=\left(G_{M},N,W_{E}\right)$$

The airspace contains a non-empty set of layers *N*, each layer being represented as a graph of nodes and airways $G_{M}=\left(V{}_{M},A{}_{M}\right)$. Nodes can belong to one or more layers.

$$V{}_{M}=\overset{\left|N\right|}{\underset{\alpha =1}{⋃}}V^{\alpha };\alpha \in N$$

Each edge, that is, airway, is assigned three different weights defining travel time, energy cost and traffic capacity respectively: $a_{M}=\left(u,v,\alpha ,t,e,c\right)$ with u,v∈$V_{M}$, α∈N and $\;t,e,c\;\in \;W_{E}$, a non-empty set of weights at event step, *E*.

The set of edges is composed of intra-layer edges, that is, airways within one layer ($A^{\alpha }$) and inter-layer edges, that is, airways connecting layers ($A^{\alpha ,\beta }$), with α,β∈ N.

$$A{}_{M}=\left(\overset{\left|N\right|}{\underset{\alpha =1}{⋃}}A^{\alpha }\right)\;⋃\;\left(\overset{\left|N\right|}{\underset{\alpha ,\beta =1,\alpha \neq \beta }{⋃}}A^{\alpha ,\beta }\right),$$

with $A^{\alpha }$⊆$V^{\alpha }\times V^{\alpha }$ and $V^{\alpha }$ a finite, non-empty set of nodes on layer α and $A^{\alpha ,\beta }$⊆$V^{\alpha }\times V^{\beta }$; with α,β∈N, α≠β and $V^{\beta }$ a finite, non-empty set of nodes on layer β.

Based on the definition in Reference [56] each layer is considered an incremental network where link weights are dynamic, that is, the structure of the network remains as is but the weights vary over time.

### 3.3. UAV Communication

**Figure 6:**
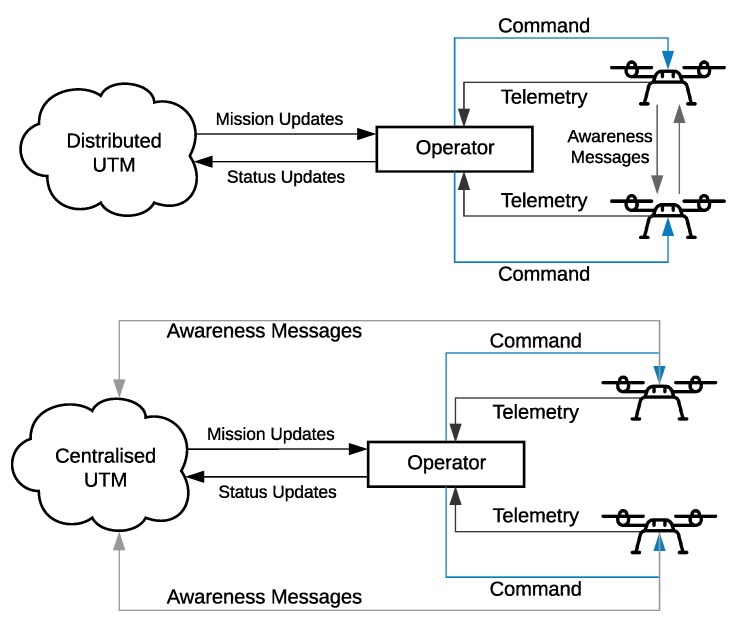
Communicated messages in a distributed and centralised UTM [15].

According to IEEE’s technical committee on networked robots [57], a wireless networked robot system (WNR) is a subset of wireless sensor and actuator networks (WSANs). Such a system can be identified by two elements: (a) autonomous capabilities and (b) network-based cooperation. The first refers to the necessity, for a robot, to autonomously move and interact with the physical environment; while the second refers to its capability of communicating with others using radio technology. Over the recent years, the interaction between IoT and flying ad-hoc networks (FANETs) has become an important topic of research [58,59]. The interested reader can find a detailed analysis of such communication protocols in References [60,61,62]. Due to the high mobility of UAVs in flight, at this stage of work, we consider UAVs to communicate together (UAV-to-UAV) and to the infrastructure (UAV-to-Infrastructure) in an ad-hoc manner, similar to the communication model proposed in References [62,63]. A simplified view of the different types of communicated messages is presented in Figure 6 based on Reference [15]. In the same article, the authors firstly categorise the types of communicated messages based on their repetition rate and size then compare the communication performance of a centralised and a distributed UTM in a conflict resolution scenario.

Here each UAV is a flying node in FANET with some acting as gateways, as illustrated in Figure 1, to relay communicated information as explained in Reference [64]. UAVs exchange information using standard IoT communication protocols [62,64] at predefined time intervals, similar to automatic dependent surveillance-broadcasts and at node locations within the network. The broadcast is composed of the UAVs’ identification, lateral flight velocity, location and timestamp. With reference to Figure 6 above, in our envisioned UTM, UAVs rely on communicated awareness messages to locally evaluate traffic conditions in airways and make routing decisions.

### 3.4. Operational Example

To consolidate the model’s description, this subsection narrates one operational example relying on the proposed multilayer model of the Class G airspace. However, our proposed model can lend itself to multiple other scenarios.

Considering two groups of IoT–enabled UAVs, the first one is on a routine surveillance mission such as the crowd-surveillance use-case discussed in Reference [58], while the second group consists of emergency intervention UAVs such as medical rescue UAVs. Both groups, equipped with different sensory payload, entering the *airspace* have different mission priorities and incentives to get to their destination. UAVs enter *airways* through different *nodes* and traverse from origin to destination along *paths* at different layers. Each altitude segment, referred to as layer, allows different velocity ranges that increase at higher altitude layers. We assume that higher altitudes offer shorter travel times at the cost of higher energy consumption.

As each UAV traverses the network, it communicates and exchanges information with other UAVs in an ad-hoc manner using standard IoT communication protocols at predefined time intervals. Based on the exchanged traffic parameters information and rules such as critical traffic density and minimum flight velocities, UAVs make local routing decisions to switch between *airways*, airspace layers and *flight modes* according to their respective objectives of minimising time of flight or energy consumption.

## 4. UAV Traffic Optimisation

Given the size of the airspace, the corresponding multilayer network might become large, making path selection NP-complete [65]. This section presents the second contribution of this work, that is the path optimisation problem formulation followed by a first approach to optimise travel time and energy separately in our model of the Class G airspace.

### 4.1. Energy-Aware Path Optimisation

We formulate the problem of minimising the total travel time and energy consumption of UAV traffic in the network. Based on our multilayer network description, the two objective functions we aim to optimise are expressed as:

(1) $$\begin{array}{cc} \min\; & P=\sum_{i=1}^{I}\sum_{l=1}^{L}a_{il}*e_{l} \end{array}$$

(2) $$\begin{array}{cc} \min\; & T=\sum_{i=1}^{I}\sum_{l=1}^{L}a_{il}*t_{l} \end{array}$$

(3) $$\begin{array}{cc} s.t.\; & \sum_{i=1}^{I}a_{il}=c_{l},\;l=1,\ldots ,L, \end{array}$$

(4) $$\begin{array}{c} c_{l}\leq c_{l}^{max},\;l=1,\ldots ,L, \end{array}$$

(5) $$\begin{array}{c} a_{il}\in \left\{0,1\right\},\;i=1,\ldots ,I,\;l=1,\ldots ,L, \end{array}$$

(6) $$\begin{array}{c} P,T\in N, \end{array}$$

(7) $$\begin{array}{c} e_{l},t_{l},c_{l}\in N,\;l=1,\ldots ,L, \end{array}$$

where:

*P*—objective function (energy consumed),
*T*—objective function (time elapsed),
*I*—number of UAVs,
*i*—index for UAVs,
*L*—number of airways,
*l*—index for airways,
*a*—selection indicator for airways/UAVs (∈0,1),
*e*—energy consumption component for airways,
*t*—time elapse component for airways,
*c*—traffic capacity for airways,
$c^{max}$—maximum traffic capacity for airways.

In our proposed model, each airway in a path has a critical traffic capacity of UAVs that it can traverse; in addition to an allowable maximum velocity which is expressed in terms of time *t* and energy *e*. Therefore, for a number of UAVs *I* over a complete path our first utility function (1) addresses our first objective, minimising the total energy consumption. While (2) addresses our second objective which is minimising the total travel time.

### 4.2. Optimisation Approach

This subsection introduces the three heuristics used in our experiments. The first one is a static path planning approach; the second is a probabilistic heuristic addressing the dynamic nature of our proposed model assuming global knowledge of the traffic conditions in the network; while the third is a pheromone guided greedy heuristic relying on local knowledge of traffic conditions. All heuristics are single objective, thus optimising either time or energy.

#### 4.2.1. Global Offline Static—UTM (GOS)

To address the static nature of the network, at the beginning, UAVs follow a pre-computed shortest path from origin to destination. This shortest path is calculated with the A* algorithm [66] using a the respective network weights *t* or *e*, depending on the minimisation objective of each UAV and a heuristic that takes into account the Euclidean distance between network nodes, assuming optimal traffic conditions, no congestion and that UAVs can traverse the network at the maximum allowable speed of the layer. UAVs follow their given path (A*_shortest_path) and update the weights t,e,c of the respective airway *l* as long as the traffic capacity on the airway, $c_{l}$ < $c_{l}^{max}$ (c.f. lines *1–4* in Heuristic 1). Once maximum capacity is reached, UAVs queue at the airway entrance node, until the condition $c_{l}$ < $c_{l}^{max}$ is satisfied (c.f. line *5* in Heuristic 1).

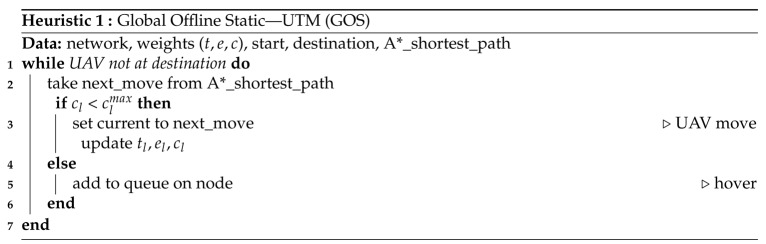



#### 4.2.2. Global Probabilistic Dynamic—UTM (GPD)

Assuming global knowledge of network weights, firstly, UAVs follow the shortest path initially computed by A* algorithm (c.f. lines *8–11* in Heuristic 2); however, on the contrary to GOS, when they encounter congestion on one airway, that is, when the maximum capacity of this airway is reached: $c_{l}$ = $c_{l}^{max}$, each UAV takes a probabilistic decision $p_{reroute}$ of either hovering in queue at the current node or to take an alternative shortest path computed with the same A* algorithm as in GOS on the multilayer network with updated t,e,c weights (c.f. lines *12–16* in Heuristic 2).

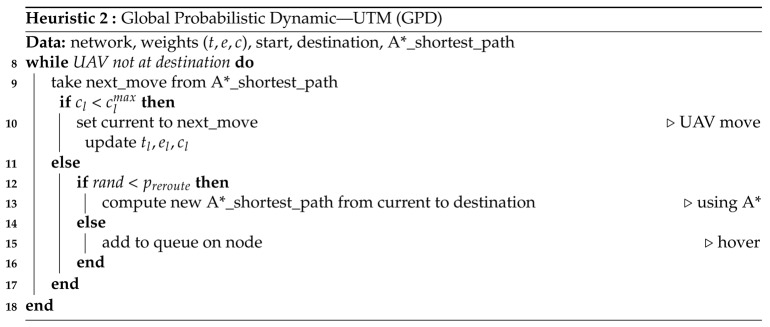



#### 4.2.3. Local Pheromone Guided—UTM (LPG)

Relying on UAVs local knowledge of traffic condition, UAVs start by following the offline generated shortest path (A*_shortest_path) similar to in GOS and GPD, until the traffic on the next airway is superior to a predefined threshold defined by $T_{lim}$, that is, when $c_{l}$ = $T_{lim}$ where $T_{lim}$ < $c_{l}^{max}$ (c.f. lines *19–22* in Heuristic 3). In reality, $T_{lim}$ would correspond to the critical traffic density explained in traffic theory as the capacity after which traffic flow becomes congested. At that stage each UAV lays down a pheromone trail τ, where $\tau_{l}$ = $1/c_{l}^{max}$ of airway *l*. The deposited trail of pheromone acts as a repellent to other UAVs, hence making the airway less desirable to take. In our model, intersecting nodes act as decision points at which the following UAVs receive the updated pheromone level and use the commonly used state transition rule introduced by Dorigo et al. in Reference [67] to decide which airway to select. This can be expressed as a function of the pheromone on the airway in addition to the quality of the airway: $p_{l}^{i}$ = $f(\tau_{l},\eta_{l})$, where $p_{l}^{i}$ is the probabilistic transition rule for UAV *i* to take airway *l* with quality $\eta_{l}$ represented by $t/c_{l}^{max}$ or $e/c_{l}^{max}$ depending on the optimisation objective of the UAV. UAVs then take a decision of either staying on the same path or selecting a new airway. If the latter, UAVs recompute a path to destination, from the newly selected airway, using A* on the multilayer network with initial weights, assuming optimal traffic conditions (c.f. lines *23–25* in Heuristic 3), otherwise UAVs remain on their initial path (c.f. lines *26–30* in Heuristic 3).

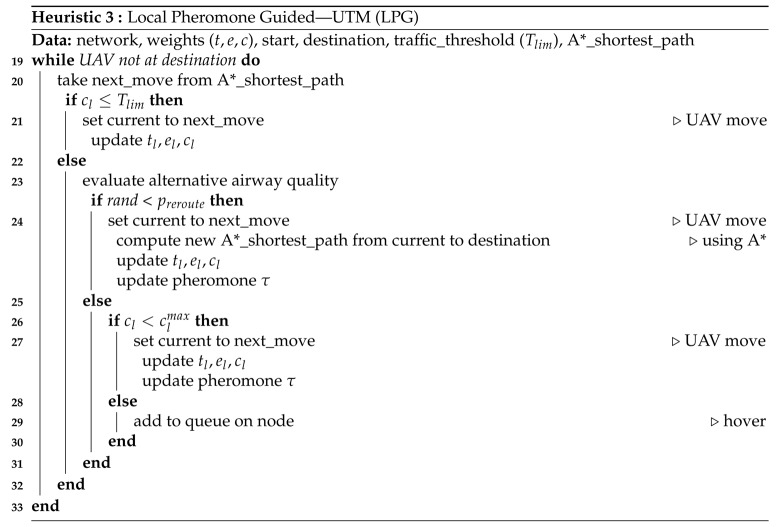



## 5. Simulation and Results

This section outlines our experimental setup and discusses the preliminary results obtained using the aforementioned three UAV traffic management heuristics.

### 5.1. Experimental Setup

**Figure 7:**
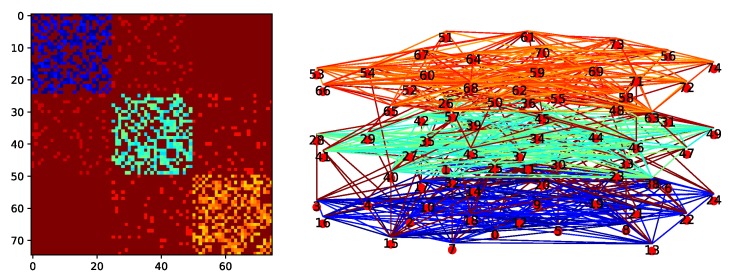
Example of a 75-Node three layer network and its adjacency matrix.

Experiments are conducted on a three layer network based on the Erdos – Rényi model using Python’s NetworkX library and the multiNetX package. Each layer contains the same number of nodes and each airway (intra and inter network) is assigned three weights, *t*, *e* and *c*, uniformly at random in predefined intervals. Figure 7 presents an example of a three layer network with 75 nodes (25 nodes per layer) and its adjacency matrix. The parameters used for the experiments are described in Table 1.

A single network with a total of 300 nodes and 3 layers (100 nodes per layer) has been used. Between every pair of nodes, there is a 20% probability an edge is created. The ranges of the three airway weights (time, energy and capacity) were selected to ensure that the lowest network layer allows less energy consumption by permitting UAVs to fly at their optimum or near optimum lateral velocity—*the velocity at which a UAV is most energy efficient benefiting from transitional lift*; while the higher layers allow incremental increase in flight velocity, hence reduce travel time at the cost of a higher energy consumption. Additionally, UAVs hovering in queuing state consume more energy than those in lateral flight. This is supported by the general power consumption model explained in Reference [68]. The selected capacity ranges also ensure that congestion can occur at different network layers for the tested UAV traffic values. Five traffic sample sizes were generated ranging from 10 to 500 UAVs (for experiment 1 and 2) and up to 1500 UAVs for experiment 3. All UAVs are assigned a pair of origin and destination nodes, both located on the lowest layer. All pairs are similar for experiment 1 and 2 while they differ in experiment 3. Each UAV keeps record of its current position, destination as well as its total travel time and energy consumption. Simulations were run 30 times for probabilistic heuristics.

Three main experiments are conducted. The first two aim to evaluate heuristics LPG and GPD respectively. For all three experiments we compare total UAVs’ travel time in the network—*in arbitrary time units*, total UAVs’ energy consumption—*in arbitrary energy units*, as well as the number of path changes, network layer change and total UAVs’ queuing counts.

The first experiment aims to investigate the effect of varying the decision probability $p_{reroute}$ of GPD on its performance. With all UAVs having the same origin and destination pairs for all 5 traffic samples, the decision probability $p_{reroute}$ is varied between 50%, 80% and 100% (c.f. Table 1), firstly with all UAVs having the same minimisation objective (i.e., time) and in a second stage with each UAV assigned either time or energy as objective.

The second experiment aims to study the effect of varying the traffic threshold $T_{lim}$ of LPG, which in turn varies the point at which UAVs start depositing pheromones on the network airways. Ensuring all UAVs have the same origin and destination pairs for all 5 traffic samples, $T_{lim}$ is varied between 0%, 50% and 80% on traffic samples with mixed minimisation objectives.

Finally, the main objective of the last experiment is to study the performance of the three heuristics (GOS, GPD, LPG) in a more realistic scenario. Each UAV is given a different pair of origin and destination and a minimisation objective (energy (1) or time (2)). The origin and destination pairs are all on the lowest layer and are more than two hops apart. The network and traffic sample size allow that airways might be shared by UAVs, hence ensuring that congestion can occur. The traffic threshold $T_{lim}$ value of LPG and decision probability $p_{reroute}$ of GPD that demonstrated best performance in the first two experiments are used.

### 5.2. Results

**Figure 8:**
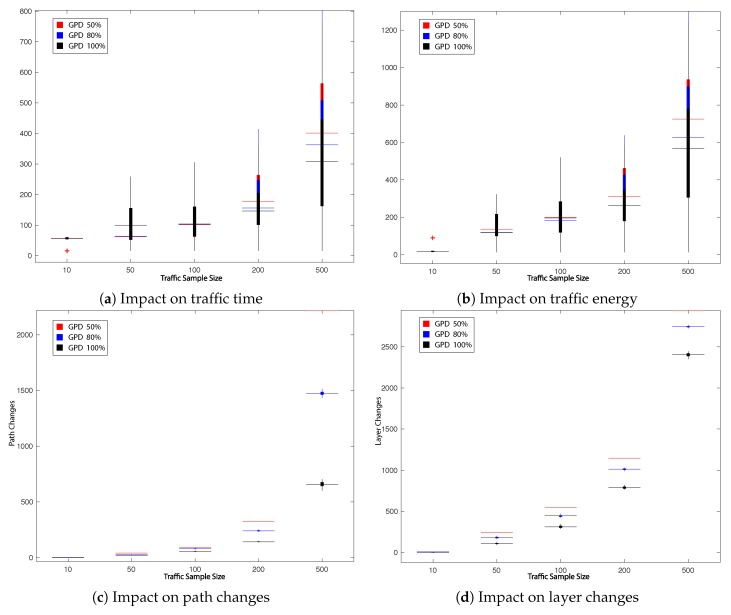
Impact on traffic performance by varying $p_{rerouting}$ in GPD (50%, 80%, 100%).

**Figure 9:**
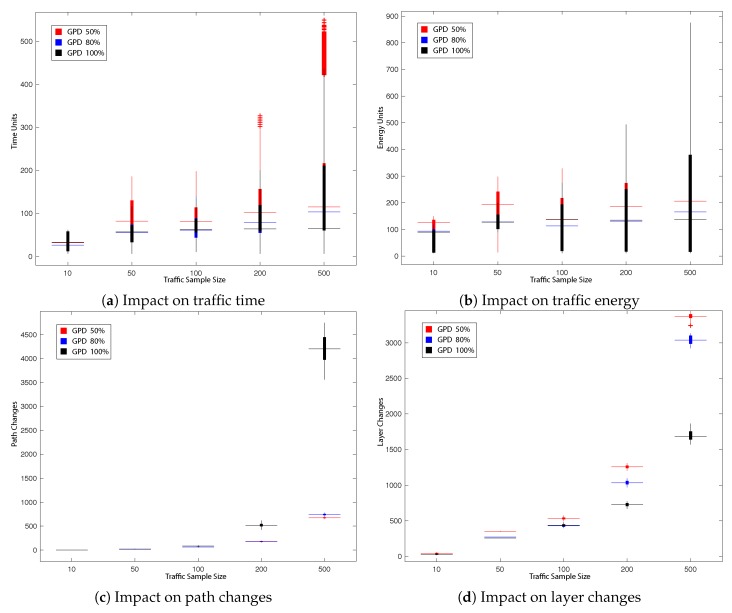
Impact on mixed objective traffic performance by varying $p_{reroute}$ in GPD (50%, 80%, 100%).

**Figure 10:**
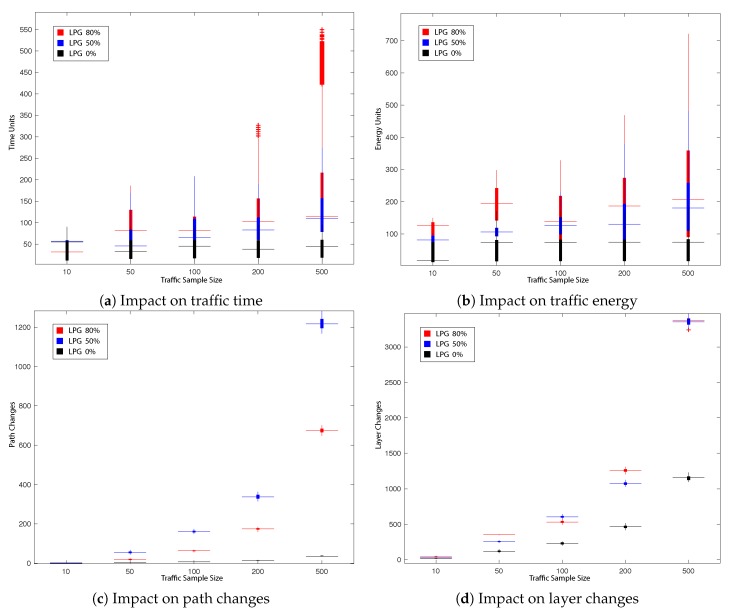
Impact on traffic performance by varying $T_{l}im$ in Local Pheromone Guided (LPG) (80%, 50%, 0%).

**Figure 11:**
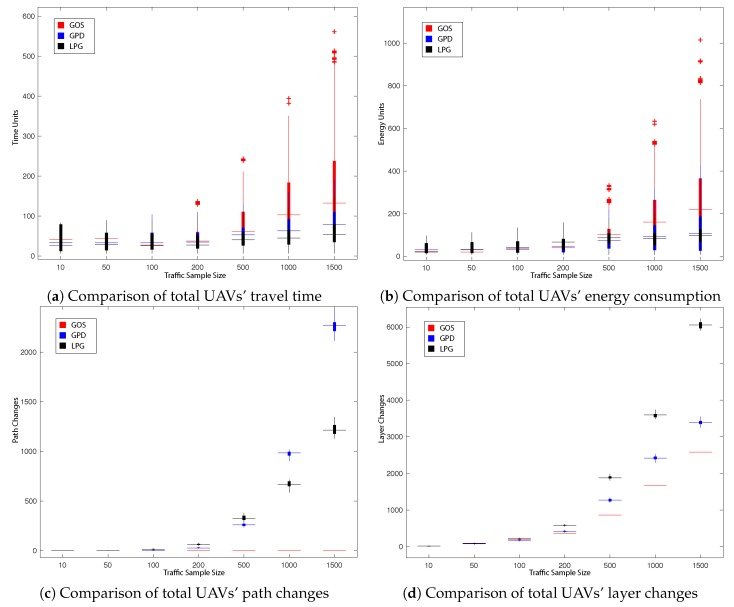
Performance comparison of Global Offline Static (GOS), GPD and LPG.

This subsection presents the main results obtained during our experiments. The impact on the total UAVs’ travel time in the network, total UAVs’ energy consumption as well as the number of path changes, network layer change and queuing/hovering counts is explored. All obtained results are presented in Figure 8, Figure 9, Figure 10 and Figure 11 and summarised in Table 2, Table 3, Table 4 and Table 5. Figure 8, Figure 9, Figure 10 and Figure 11 present the impact in traffic performance by indicating the median, 25th and 75th percentile, while Table 2, Table 3, Table 4 and Table 5 present the mean and standard deviation in the results after 30 runs of the probabilistic heuristics for every varied parameter over all traffic samples: for every $T_{lim}$ in LPG and for every $p_{rerouting}$ in GPD. Statistical confidence in our comparisons is assessed by performing Kruskal-Wallis test [69] for Experiments 1 and 2 respectively and by performing the Wilcoxon test [70] for Experiment 3. The overall best result per comparison parameter is shown in bold. Additionally, the dark grey background emphasises the best results that showed statistically significant difference with a 95% confidence.

#### 5.2.1. Experiment 1: Impact of $p_{rerouting}$ on GPD Performance

In the first experiment we aim to study the impact of the decision probability $p_{reroute}$ on the performance of GPD, firstly, for traffic samples consisting of UAVs with the same minimisation objective, then with traffic samples with varying minimisation objectives. Three $p_{reroute}$ values are tested as indicated in Table 1: 50%, 80% and 100%.

It can be deduced from Table 2 and Figure 8, of the first part of the experiment, that for the smaller traffic sample sizes of 10 and 50, GPD with $p_{reroute}$ 50% and 80% generally showed improvement over $p_{reroute}$ of 100% in total UAVs’ travel time and energy consumption. Nevertheless, $p_{reroute}$ of 100% showed better performance when it came to total traffic path and layer changes for the same samples. However, since the main global objective is a scalable system, it is important to study the performance of the heuristic at larger sample sizes. While that is the case, GPD with $p_{reroute}$ of 100% outperforms the same heuristic with $p_{reroute}$ 50% and 80% for the larger traffic samples. Improvements can be observed with statistically significant difference across all the traffic performance indicators tested for traffic sample size 500 and 200, with exception of total UAVs’ queue counts in the network for the latter.

A similar trend is observed in the second part of the experiment, presented in Table 3 and Figure 9 where UAVs have different minimisation objectives as explained in Section 5.1.

Compared to the first part, Table 3 and Figure 9 showed a significant improvement for the total UAVs’ travel time and energy consumption for GPD with $p_{reroute}$ 100% for larger traffic samples, regardless of the individual minimisation objective of UAVs.

#### 5.2.2. Experiment 2: Impact of $T_{lim}$ on LPG Performance

Similar to Experiment 1 (Section 5.2.1), the impact on total UAVs’ travel time and energy consumption in the network as well as the number of path changes, network layer change and queuing counts is explored as result of varying traffic threshold $T_{lim}$ of LPG between 0%, 50% and 80% of $c_{l}^{max}$.

Analysing the results obtained, it can be deduced from Table 4 and Figure 10 that, with the exception for the smallest traffic sample size of 10, LPG with $T_{lim}$ of 0% generally showed improvement with statistically significant difference over $T_{lim}$ of 80% and 50% across all the traffic performance indicators tested for the remaining traffic sample sizes. These results indicate that pheromone deposit caused a fast dispersion of UAVs across the network, which had a negative impact only for the smallest traffic sample. On the contrary, it led to an overall improvement in reducing total UAVs’ travel time and energy consumption across for all other traffic samples.

#### 5.2.3. Experiment 3: Performance Comparison of GOS, GPD and LPG

Finally, Experiment 3 aims to study the performance of the three heuristics (GOS, GPD, LPG) in a more realistic scenario as explained in Section 5.1 to address the assumptions made in the previous work in the literature [22]. Here each UAV has a different origin and destination pair as well as one of the two minimisation objectives (energy (1) or time (2)).

Figure 11 and Table 5 present the obtained results when comparing the impact the three heuristics (GOS, GPD, LPG) have on traffic performance in a more realistic scenario. It can be observed that, with the exception for traffic sample 10, LPG results show improvement in total UAVs’ travel time for all traffic samples, where the percentage of UAVs with the minimisation objective (2) (i.e., time) is 50%, 40%, 45%, 19.5%, 48.8%, 49% and 48.13% for every traffic sample size respectively. On the other hand, it is worth to mention that due to the selected parameters and the nature of GPD, encouraging UAVs to be more inclined to reduce layer changes, led to the significant difference in reduction of energy consumption in comparison to LPG for traffic samples 50–200. However, for the larger traffic samples, which are more decisive in the devised scenario, LPG outperforms GPD with significant difference across 4 of the 5 main parameters of comparison, with the exception of total number of layer changes, which can be explained by the nature of the heuristic LPG which encourages UAVs to explore vertical airways between layers as they offer a higher $c_{l}^{max}$.

## 6. Conclusions and Future Work

IoT has catalysed and facilitated the development of new devices that are capable of interacting with our physical world, generating unprecedented amounts of information. One category that not only promise a new means of capturing information but also new means of overcoming limitations of IoT’s existing infrastructure are UAVs. However, despite the significant advantages UAVs bring, as their number continues to grow, UAV deployment is faced with challenges in their safe operation and management. This emphasises the need for a new dedicated, distributed UAV traffic management system supported by regulations and international technical standards.

In this paper, we have extended our first contributions towards our envisioned distributed UTM by presenting a broader state of the art analysis as well as description of UAV communication. The work, firstly, described our model and structure of the low altitude, Class G, airspace and outlines key terminology and an operational scenario. Secondly, the paper contributed to existing literature by representing the airspace model as a multilayer network of nodes and airways. Finally the corresponding UTM optimisation problem has been defined and some first experimental results have been obtained using three traffic management heuristics. The results showed that for the larger traffic samples, the heuristic assuming local traffic knowledge, LPG, outperforms GOS and GPD across the main traffic performance indicators selected for the study.

Whilst the simulation included assumptions to simplify the challenging multifaceted problem, future work will focus on more realistic communication scenarios. We intend to investigate different communication protocols on traffic behaviour and explore more realistic communication metrics, given the challenging nature of flying ad-hoc networks. Finally, we intend to develop and evaluate different metaheuristic optimisation techniques for this challenging problem.

## Figures and Tables

**Table 1:** Experiment parameters.

Parameter	Value
Number of UAVs (*experiment 1 & 2*)	10, 50, 100, 200, 500
Number of UAVs (*experiment 3*)	10, 50, 100, 200, 500, 1000, 1500
Number of nodes	100 per layer
Number of layers	3
Edge creation probability	20%
Interlayer energy weight interval	[15,20]
Intralayer energy weight intervals	[5,10], [15,20], [25,30]
Interlayer time weight interval	[1,5]
Intralayer time weight intervals	[25,30], [15,20], [5,10]
Interlayer capacity weight interval	50
Intralayer capacity weight interval	[1,5]
GPD decision probability ($p_{reroute}$)	50%, 80%, 100%
LPG $T_{lim}$ percentage of $c_{l}^{max}$	0%, 50%, 80%

**Table 2:** Impact on traffic performance from varying $p_{reroute}$ in Global Probabilistic Dynamic (GPD) (50%, 80%, 100%).

Traffic	$p_{reroute}$	Time	Energy	Path Changes	Layer Changes	Queue Counts
		Mean_SD_	Mean_SD_	Mean_SD_	Mean_SD_	Mean_SD_
**10**	50%	**52.9_12.562_**	**24.3_22.073_**	1_0_	6_0_	**0_0_**
	80%	55.22_13.532_	24.913_24.122_	0.733_0.442_	4.4_2.657_	0.267_0.442_
	100%	59.57_14.195_	26.063_27.518_	**0.233_0.423_**	**1.4**_2.541_	0.767_0.423_
**50**	50%	72.58_32.991_	126.06_60.396_	41_0_	246_0_	**0_0_**
	80%	**71.609_31.663_**	**113.490_52.779_**	31.1_1.578_	180.533_12.631_	14.067_4.7127_
	100%	95.709_54.985_	142.730_85.650_	**19.4_2.260_**	**108.6_13.237_**	46.767_6.683_
**100**	50%	101.54_44.173_	185.78_77.196_	91_0_	546_0_	**41_0_**
	80%	**97.807_41.408_**	**168.034_71.125_**	80.667_3.123_	444.8_11.975_	53.067_1.999_
	100%	119.549_68.633_	203.498_112.434_	**55.533_2.391_**	**311.4_14.881_**	132.4_13.439_
**200**	50%	184.77_98.680_	321.89_152.576_	323_0_	1146_0_	273_0_
	80%	174.292_98.3507_	293.762_153.836_	241.2_4.942_	1012.2_9.789_	**255.767_2.362_**
	100%	**155.051_79.231_**	**274.623_138.601_**	**142.333_3.261_**	**790.667**_15.086_	344.7_15.775_
**500**	50%	403.158_218.089_	692.006_359.752_	2219_0_	2946_0_	2169_0_
	80%	362.4707_200.818_	626.507_340.444_	1474.867_20.884_	2746_8.884_	2002.067_9.609_
	100%	**317.376**_175.792_	**560.534**_297.477_	**660.667**_23.310_	**2403.533**_23.408_	**1932.6**_5.897_

**Table 3:** Impact on mixed objective traffic performance from varying $p_{\mathrm{reroute}}$ in GPD (50%, 80%, 100%).

Traffic	$p_{\mathrm{reroute}}$	Time	Energy	Path Changes	Layer Changes	Queue Counts
		Mean	Mean	Mean	Mean	Mean
**10**	50%	36.983_16.054_	81.483_59.709_	0.567_0.668_	44_6.120_	**0**_0_
	80%	**34.693**_18.101_	64.133_41.679_	0.7_0.972_	34_6.461_	**0**_0_
	100%	35.29_23.037_	**55.49**_42.019_	**0.066**_0.249_	**30.4**_8.346_	**0**_0_
**50**	50%	90.256_48.051_	184.011_76.146_	19.033_3.231_	353.267_3.327_	0.1_0.300_
	80%	56.010_23.587_	122.049_42.648_	20.233_3.364_	270_3.347_	0.2_0.541_
	100%	**53.612**_25.564_	**119.072**_49.721_	**17.4**_3.989_	**258**_2.858_	**0**_0_
**100**	50%	87.818_45.792_	143.507_90.842_	63.8_2.982_	531.867_18.179_	6_2.161_
	80%	67.753_28.558_	**105.883**_55.172_	**62.4**_4.580_	**431.8**_20.706_	**4.8**_1.939_
	100%	**67.037**_27.559_	127.385_78.674_	82.967_6.868_	439.4_15.928_	22.233_2.654_
**200**	50%	113.013_61.736_	188.479_113.829_	**174.5**_6.329_	1259.2_28.304_	17.1_4.962_
	80%	97.522_66.314_	153.513_139.743_	182.067_5.994_	1034.6_29.251_	**16.567**_4.064_
	100%	**79.363**_30.984_	**133.671**_67.384_	517.5_36.862_	**731.867**_29.662_	90.233_7.196_
**500**	50%	160.941_115.246_	239.410_170.669_	**673.9**_12.081_	3372.933_46.131_	548.033_44.961_
	80%	149.9787_130.437_	232.054_252.268_	745.733_17.309_	3040.2_60.176_	39.867_6.265_
	100%	**118.104**_63.245_	**180.317**_108.839_	4177.1_300.999_	**1705.067**_80.250_	**38.633**_6.264_

**Table 4:** Impact on traffic performance from varying traffic threshold in LPG (80%, 50%, 0%).

Traffic	$T_{lim}$	Time	Energy	Path Changes	Layer Changes	Queue Counts
		Mean_SD_	Mean_SD_	Mean_SD_	Mean_SD_	Mean_SD_
**10**	80%	**35.897**_16.054_	81.483_59.709_	0.567_0.669_	44_6.120_	**0**_0_
	50%	36.983_21.059_	60.211_38.824_	2.167_0.523_	26.333_2.199_	**0**_0_
	0%	41.048_22.902_	**42.883**_31.621_	**0.033**_0.179_	**19.2**_5.5131_	0.033_0.179_
**50**	80%	90.256_48.051_	184.011_76.146_	19.033_3.231_	353.267_3.327_	0.1_0.303_
	50%	58.083_34.847_	104.555_38.793_	56.2_6.597_	256.4_8.788_	**0.033**_0.179_
	0%	**38.535**_23.058_	**60.263**_46.212_	**3.767**_0.558_	**118.267**_13.177_	3.767_0.558_
**100**	80%	87.8175_45.792_	143.507_90.842_	63.8_2.982_	531.867_18.179_	6_2.165_
	50%	82.312_55.216_	138.027_68.833_	161.767_6.855_	604.067_17.257_	**0.667**_0.788_
	0%	**39.929**_22.709_	**59.784**_46.062_	**7.2**_0.653_	**230**_17.400_	7.2_0.653_
**200**	80%	113.014_61.736_	188.479_113.829_	174.5_6.329_	1259.2_28.303_	17.1_4.962_
	50%	91.602_50.154_	134.861_81.634_	338.967_14.102_	1079.4_31.008_	**3.867**_1.857_
	0%	**40.282**_22.734_	**60.974**_46.324_	**14.867**_1.118_	**465.733**_26.967_	14.867_1.117_
**500**	80%	160.942_115.246_	239.411_170.669_	673.9_12.0816_	3372.933_46.131_	38.633_6.263_
	50%	131.893_85.076_	191.934_122.321_	1218.333_37.382_	3355.667_51.543_	**35.6**_6.988_
	0%	**40.798**_22.697_	**60.925**_46.422_	**37.067**_1.672_	**1154**_33.765_	37.067_1.672_

**Table 5:** Comparison of traffic performance using GOS, GPD and LPG.

Traffic	Heuristic	Time	Energy	Path Changes	Layer Changes	Queue Counts
		Mean_SD_	Mean_SD_	Mean_SD_	Mean_SD_	Mean_SD_
**10**	GOS	**36.6**_24.88_	**33.2**_22.836_	**0**_0_	**16**_0_	**0**_0_
	GPD	37.005_27.297_	37.468_25.579_	**0**_0_	17.933_7.006_	**0**_0_
	LPG	41.48_28.825_	34.636_24.895_	**0**_0_	15.467_5.142_	**0**_0_
**50**	GOS	42.48_24.708_	**36.12**_25.274_	**0**_0_	**80**_0_	**0**_0_
	GPD	40.075_27.126_	38.069_26.758_	0.2_0.603_	84.6_12.759_	**0**_0_
	LPG	**38.567**_26.827_	39.741_27.149_	1.267_1.367_	92_12.365_	**0**_0_
**100**	GOS	38.96_25.905_	42.75_27.849_	**0**_0_	196_0_	0_0_
	GPD	40.551_26.048_	**40.884**_28.378_	2.133_1.707_	**175.667**_13.811_	0.167_0.453_
	LPG	**36.05**_24.306_	47.796_29.8793_	13.6_4.957_	228.733_8.982_	**0**_0_
**200**	GOS	46.755_31.381_	52.42_35.264_	**0**_0_	**360**_0_	17_0_
	GPD	41.475_25.571_	**51.0127**_32.667_	27.8_4.527_	420.6_15.512_	4.767_2.458_
	LPG	**34.823**_21.419_	59.737_31.569_	63.167_6.798_	585.067_16.426_	**0**_0_
**500**	GOS	80.355_53.447_	109.591_73.879_	**0**_0_	**864**_0_	300_0_
	GPD	54.502_32.649_	**82.0328**_59.460_	258.333_20.190_	1275_38.084_	101.433_10.941_
	LPG	**45.676**_25.424_	84.797_41.121_	331_24.960_	1895.533_49.837_	**1.1**_1.247_
**1000**	GOS	123.007_84.891_	189.113_131.816_	**0**_0_	**1668**_0_	1372_0_
	GPD	76.069_50.318_	111.649_101.295_	982.433_40.802_	2425.933_54.944_	441.467_25.967_
	LPG	**49.867**_27.228_	**82.845**_42.786_	670.5_33.059_	3592.267_57.933_	**10.433**_4.318_
**1500**	GOS	162.340_114.463_	264.691_193.237_	**0**_0_	**2584**_0_	3097_0_
	GPD	93.355_69.075_	137.715_142.167_	2267.9_81.103_	3400.933_75.662_	1059.033_66.985_
	LPG	**59.887**_34.968_	**100.8399**_53.280_	1220.533_52.787_	6051.533_96.466_	**11.8**_4.490_
